# Dynamics of body composition in the treatment of locally advanced rectal cancer: the ımpact of adipose and muscle tissue on pathological response

**DOI:** 10.1590/1806-9282.20250752

**Published:** 2025-10-27

**Authors:** Okan Dilek, Emin Demırel, Berkay Eyı, Seyda Gokce Turunc, Huseyin Akkaya, Zeynel Abidin Tas, Görkem Ozdemır, Timuçin Cıl, Gokhan Soker

**Affiliations:** 1 University of Health Sciences, Adana City Training and Research Hospital, Department of Radiology – Adana, Turkey.; 2 Afyonkarahisar University of Health Sciences, Faculty of Medicine, Department of Radiology – Afyonkarahisar, Turkey.; 3 Ondokuz Mayis University, Faculty of Medicine, Department of Radiology – Samsun, Turkey.; 4 University of Health Sciences, Adana Teaching and Research Hospital, Department of Pathology – Adana, Turkey.; 5 University of Health Sciences, Adana Teaching and Research Hospital, Department of Gastroenterological Surgery – Adana, Turkey.; 6 University of Health Sciences, Adana Teaching and Research Hospital, Department of Medical Oncology – Adana, Turkey.

**Keywords:** Rectal cancer, Neoadjuvant chemoradiotherapy, Neoplasm regression, Body composition, Psoas muscles

## Abstract

**OBJECTIVE:**

The aim of the study was to evaluate the role of body composition parameters measured via computed tomography before and after neoadjuvant chemoradiotherapy in predicting pathological response in patients with locally advanced rectal cancer.

**METHODS:**

Eighty-four patients with locally advanced rectal cancer who underwent neoadjuvant chemoradiotherapy followed by curative surgery were retrospectively analyzed. Computed tomography images obtained before and after neoadjuvant chemoradiotherapy were used to assess total adipose area, visceral adipose tissue, subcutaneous adipose tissue, adipose tissue density, psoas muscle area, psoas muscle density, and mesorectal adipose volume and density at the level of the L3 vertebra. Pathological response was determined using the Ryan Tumor Regression Grade system. Baseline values and percentage changes (Δ) during treatment were compared between response groups.

**RESULTS:**

Of the 84 patients, 31 (36.9%) showed a pathological response. There were no significant differences in demographic or clinical features between the groups. Among baseline body composition metrics, only psoas muscle density was significantly higher in the response group (p=0.015). Post-treatment analysis revealed a significant difference only in the percentage change in psoas muscle density (ΔPMD) (p=0.023). No other baseline or percentage change values for total adipose area, subcutaneous adipose tissue, visceral adipose tissue, mesorectal adipose volume, or psoas muscle area were significantly associated with pathological response (p>0.05).

**CONCLUSION:**

In patients with locally advanced rectal cancer, neither abdominal adipose composition nor mesorectal adipose volume nor psoas muscle area was predictive of response to neoadjuvant chemoradiotherapy. However, psoas muscle density, a potential indicator of muscle quality, may serve as a promising biomarker for predicting pathological response based on both baseline value and treatment-induced changes.

## INTRODUCTION

Rectal cancer constitutes about one-third of colorectal cancers and is frequently diagnosed at an advanced stage^1^. Together, colorectal cancers are the third most common globally and the second leading cause of cancer-related death^2^. Standard treatment for locally advanced rectal cancer (LARC) involves neoadjuvant chemoradiotherapy (nCRT) followed by total mesorectal excision (TME)^3,4^. nCRT has been shown to reduce local recurrence and improve overall and disease-free survival^5^. However, only 10–30% of patients achieve a pathological complete response (pCR), and response varies significantly^6^. Due to this variability and low pCR rates, a multidisciplinary approach is needed to identify predictive factors, including potential links between post-nCRT changes in muscle/adipose mass and pathological response^7^.

Body composition, assessed via skeletal muscle and adipose areas at the L3 level on computed tomography (CT), serves as a biomarker of inflammation and surgical risk and guides personalized treatment. Muscle and fat tissues impact oncologic outcomes by affecting metabolic and immune responses. In LARC, mesorectal fat affects radiotherapy response, while overall muscle and fat mass may influence nCRT outcomes^8,9^. Some studies support these as predictors, while others do not^7,10^. Nutritional status, increasingly recognized for its role in cancer outcomes^11^, is better assessed by body composition than by body mass index (BMI), which lacks sensitivity to sex differences and fat distribution. Despite extensive research, no consistent biomarker has been established for routine use. This study aims to evaluate whether changes in mesorectal fat, abdominal adipose tissue, and muscle mass predict pCR after nCRT in LARC patients.

## METHODS

### Patient selection

This study has been approved by the Ethics Committee (11-2025) and was conducted in full accordance with the guidelines of the Helsinki Declaration. A retrospective review was conducted of 370 patients diagnosed with rectal cancer at our hospital between January 1, 2017, and December 31, 2024. A total of 84 patients who underwent pre-treatment colonoscopic biopsy and had a pathological diagnosis of rectal cancer were included in the study. Demographic data such as age, gender, height, weight, BMI, and medical history were obtained from the hospital information services database. Local staging of patients was performed using magnetic resonance imaging (MRI), with T and N assessment criteria. Lymph nodes with a diameter greater than 5 mm or those with an unfavorable morphology were pathologically evaluated for lymph node involvement. N0 represented no lymph node involvement, N1 indicated 1–3 suspicious nodes, and N2 indicated four or more suspicious nodes. To evaluate distant organ metastases, thoracoabdominal CT scans were performed.

### Inclusion criteria

- Histopathologically confirmed rectal adenocarcinoma via pre-nCRT colonoscopic biopsy.
- Clinically or radiologically diagnosed LARC (cT3–4 and/or cN+) based on pre-nCRT pelvic MRI and/or thoracoabdominal CT.
- Completion of standard nCRT.
- Underwent radical surgery (TME) after nCRT.
- Availability of high-quality pre- and post-nCRT abdominal CT scans suitable for body composition analysis.

### Exclusion criteria

- History of other primary malignancies or concomitant cancers.
- Presence of metastatic disease.
- CT artifacts from metallic implants (e.g., spinal hardware) obstruct body composition assessment at the L3 level.
- Previous rectal surgery, pelvic radiotherapy, or abdominal surgery.
- Massive ascites on pre- or post-nCRT CT scans.
- Non-operative management due to complete clinical response after nCRT ("watch and wait" approach).
- Missing clinical, pathological, or imaging data.

### Neoadjuvant chemoradiotherapy treatment and pathological response

All patients received the same nCRT regimen. The decision to initiate nCRT or proceed directly to radical resection was made by a multidisciplinary team comprising surgeons, oncologists, pathologists, and radiologists. Radiation therapy, delivered in 25 fractions, ranged from 45 to 50 Gy and followed institutional protocols. Concurrently, patients received oral capecitabine at a daily dose of 1,650 mg/m^2^.

Pathological staging was performed according to the 8th edition of the American Joint Committee on Cancer (AJCC) guidelines, as recommended by the National Comprehensive Cancer Network (NCCN). Postoperative pathological response was evaluated by a designated pathologist to ensure standardized assessment. The modified Ryan tumor regression grading system was used to classify response^12^, defining it as:

- Complete response: no viable cancer cells,
- Near-complete response: rare or few residual tumor cells,
- Minimal response: slight regression of tumor cells,
- No response: no histological regression.

For analysis, patients with complete or near-complete response were grouped as response-positive, while those with minimal or no response were classified as response-negative.

### Measurement and definition of body composition

Imaging was performed using a 128-detector multidetector computed tomography (MDCT) system (Philips Ingenuity 128, Eindhoven, Netherlands). The technical parameters used were: 120 kVp, 200–400 mAs with automatic tube current modulation, rotation time 0.42 s, pitch 0.6, and slice thickness: 1 mm for all phases. Contrast-enhanced CT images of the patients were uploaded to the open-source, web-based "CoreSlicer" tool (https://coreslicer.com/). Measurements of total adipose tissue area (TFA), visceral adipose tissue area (VAT), subcutaneous adipose tissue (SAT), psoas muscle area (PMA), and psoas muscle density (PMD) were automatically performed. Mesorectal adipose volume (MRV) was measured using the 3DSlicer method (https://www.slicer.org/). Measurements were performed by two radiologists with 2 and 12 years of experience.

### Statistical methods

The Kolmogorov-Smirnov test was used to determine the normality of the data. Normally distributed data were expressed as means±standard deviations and analyzed using an independent sample t-test, while skewed data were expressed as medians (interquartile ranges) and analyzed using the Mann-Whitney U test. Categorical variables were analyzed using the chi-square test or Fisher's exact test. The percentage change (Δ) for each body composition parameter (TFA, SAT, VAT, MRV, PMA, PMD) during nCRT was calculated using the following formula:

ΔParameter (%)=[(post-nCRT parameter - pre-nCRT parameter)/pre-n nCRT parameter]×100

A p<0.05 was considered statistically significant.

## RESULTS

Out of the 84 patients included in the study, 31 (36%) showed a pathological response to nCRT, while 53 (64%) did not. There were no significant differences in age, gender, T stage, nodal involvement, tumor localization, or BMI between the groups with and without pathological response. Detailed demographic and tumor stage-related data are presented in Table 1.

**Table 1:** Detailed information regarding demographic data and tumor staging is provided in the table.

		pCR (n=31)	non-pCR (n=53)	p
		Mean±SD/n%	Mean±SD/n%	
Age (years)		62.4±12.4	63.6±11.3	0.652
Gender				
	Female	12 (38%)	23 (43%)	0.674
	Male	19 (62%)	30 (57%)	
T stage				
	T2	11 (35%)	11 (20%)	0.311
	T3	18 (58%)	39 (73.5%)	
	T4	2 (7%)	3 (6.5%)	
Node				
	N0	4 (12%)	7 (13%)	0.985
	N1	19 (61%)	31 (58.5%)	
	N2	8 (27%)	15 (28.5%)	
Location				
	Proximal	9 (29%)	15 (28.3%)	0.756
	Middle	16 (51%)	23 (43.4%)	
	Distal	4 (13%)	13 (24.5%)	
	Diffuse	2 (7%)	2 (3.8%)	
	BMI	25.01±8.8	26.8±5.7	0.485

pCR: pathological complete response; SD: standard deviation; BMI: body mass index.

In LARC patients, there were significant differences only in PMD (p=0.015) between the pathological response-positive and negative groups when comparing BMI, TFA, SAT, VAT, MRV, PMA, and densities before and after nCRT. Detailed data are shown in Table 2. When comparing the differences in TFA, SAT, VAT, MRV, PMA, and densities before and after treatment, a significant difference was found only between the PMD groups (p=0.023). Further details are presented in Table 3.

**Table 2:** Body adipose and muscle tissue mass and densities before and after treatment are shown.

	pCR (n=31)	non-pCR (n=53)	P
	Mean±SD	Mean±SD	
Pre-NCRT, total adipose area (cm^2^)	390.1±194	338.1±202	0.252
Pre-NCRT, total adipose density (HU)	(-87.2)±11	(-87.5)±10.4	0.990
Post-NCRT, total adipose area (cm^2^)	370±175.2	325.8±190.5	0.294
Post-NCRT, total adipose area (HU)	(-87.0)±10.5	(-85.5)±10.9	0.530
Pre-NCRT, subcutaneous adipose area (cm^2^)	216.1±120.4	193.3±138.2	0.441
Pre-NCRT, subcutaneous adipose density (HU)	(-92.2)±9.6	(-90)±14.1	0.442
Post-NCRT, subcutaneous adipose area (cm^2^)	203.2±107	185±124.5	0.495
Post-NCRT, subcutaneous adipose density (HU)	(-91.2)±10.4	(-90)±11.3	0.692
Pre-NCRT, visceral adipose area (cm^2^)	173.9±107.7	145±87.7	0.187
Pre-NCRT, visceral adipose density (HU)	(-82.2)±15.2	(-84.5)±12.4	0.447
Post-NCRT, visceral adipose area (cm^2^)	166.6±101.7	140.8±90.9	0.228
Post-NCRT, visceral adipose density (HU)	(-83)±11	(-80.9)±12	0.892
Pre-NCRT, mesorectal adipose volume (cm^3^)	121±52	129.1±50.2	0.430
Pre-NCRT, mesorectal adipose fat density (HU)	(-58.7)±14.3	(-51.3)±17.5	0.051
Post-NCRT, mesorectal adipose volume (cm^3^)	100.8±48	105.5±55.7	0.693
Post-NCRT, mesorectal adipose density (HU)	(-52.2)±15.7	(-47.4)±20	0.179
Pre-NCRT, mean psoas muscle area (cm^2^)	8.4±3.1	7.8±2.9	0.980
Pre-NCRT, mean psoas muscle density (HU)	39.5±9.2	36.3±7.9	0.015
Post-NCRT, mean psoas muscle area (cm^2^)	8.5±2.9	7.5±2.8	0.706
Post-NCRT, mean psoas muscle density (HU)	36.8±8.4	38.1±7.6	0.898

pCR: pathological complete response; SD: standard deviation; NCRT: neoadjuvant chemoradiotherapy.

**Table 3:** Changes in adipose and muscle tissue mass and densities after neoadjuvant chemoradiotherapy are shown.

	pCR (n=31)	non-pCR (n=53)	p
ΔTotal adipose area (cm^2^)	(-0.06)±0.180	0.023±0.640	0.636
ΔTotal adipose density (HU)	0.008±0.146	(-0.019)±0.081	0.282
ΔSubcutaneous adipose area (cm^2^)	(-0.048)±0.135	0.015±0.309	0.288
ΔSubcutaneous adipose density (HU)	(-0.012)±0.070	0.020±0.179	0.344
ΔVisceral adipose area (cm^2^)	(-0.008)±0.216	(-0.016)±0.390	0.177
ΔVisceral adipose density (HU)	0.131±0.802	0.037±0.124	0.135
ΔMesorectal adipose volume (cm^3^)	(-0.177)±0.354	(-0.175)±0.275	0.407
ΔMesorectal adipose density (HU)	(-0.070)±0.246	0.235±0.643	0.441
ΔMean psoas muscle area (cm^2^)	0.034±0.172	(-0.085)±0.161	0.168
ΔMean psoas muscle density (HU)	(-0.050)±0.183	0.090±0.304	0.023

pCR: pathological complete response.

## DISCUSSION

In this retrospective study, we comprehensively investigated the role of CT-based body composition parameters in predicting pathological response to standard nCRT in LARC patients, considering both baseline values and changes during treatment. The most striking finding in our study was that PMD, a marker of muscle quality, showed a significant relationship with pathological response. Patients with higher baseline PMD and those who preserved PMD better during nCRT (i.e., maintained better muscle quality) were more likely to show a favorable pathological response. In contrast, the quantity of muscle mass (PMA) and various adipose tissue compartments (TFA, SAT, VAT, MRV) or changes in these parameters did not serve as significant markers for predicting pathological response in our cohort.

In our study, there was no statistically significant difference between pathological response and age, gender, tumor T stage, nodal stage, tumor localization, and BMI at diagnosis, indicating that these factors did not have a direct impact on treatment response. Literature shows mixed results regarding the relationship between T stage, tumor localization, BMI, and nCRT response, with some studies suggesting a potential predictive value, while others found no such association^7,13-15^. In locally advanced tumors, nCRT does not have the same effect on every patient, and individual differences play a crucial role in treatment outcomes. Therefore, we believe that factors beyond the tumor also affect treatment response.

Our results contribute to the mixed findings in the literature regarding the relationship between adipose tissue amounts (TFA, SAT, VAT, MRV) and pathological response. Some studies suggest that visceral obesity or high MRV may be associated with poor response or increased surgical complications, while our findings and those of other studies did not confirm this relationship^7,16^. The inflammatory and metabolic effects of adipose tissue are complex, and it is possible that functional characteristics, such as adipokine profiles, rather than the mere quantity of adipose tissue, may be more closely related to treatment response.

Sarcopenia has been suggested as a predictor of pathological response to nCRT in some studies, while other studies have shown no predictive value^17,18^. In our study, no significant difference was observed between patients with and without nCRT response in terms of muscle mass. However, when assessing muscle density, both pre-treatment and post-treatment changes in density seemed to offer a promising way to predict treatment response. Higher initial PMD (less myosteatosis) and better preservation of PMD during treatment were associated with a favorable pathological response. This suggests that not only the quantity but also the quality of muscle may play a critical role in treatment response. High muscle density is an indirect indicator of low intramuscular adipose and suggests healthy muscle tissue. The mechanisms underlying myosteatosis are not fully understood, but it is known to be associated with increased local and systemic inflammation, insulin resistance, mitochondrial dysfunction, and reduced muscle protein synthesis^19^. These conditions may adversely affect chemotherapy metabolism and efficacy, radiation therapy cellular response, and overall treatment tolerance.

According to the literature, high muscle density has been shown to positively correlate with survival in colorectal cancer patients^19,20^. However, studies specifically investigating the effect of changes in muscle density during nCRT in rectal cancer patients are limited. Therefore, pre-treatment PMD and its change post-treatment may be an important prognostic marker, warranting larger-scale and prospective studies for direct clinical application.

There are several limitations to this study. First, it is a single-center retrospective study, which inevitably introduces selection bias. Second, due to the relatively small sample size, some research endpoints related to body composition showed only trends but did not achieve statistical significance. Third, this study only examined body composition at two time points (pre- and post-nCRT), and we did not track the longer-term effects of body composition changes on patient prognosis. Fourth, we do not have detailed information on the patients’ molecular mutation profiles, which represents another limitation in the interpretation of the results.

## CONCLUSION

In conclusion, we found that PMD, rather than body adipose parameters, is a potential predictor of pathological response to nCRT in LARC patients. Our results suggest that the quality of muscle tissue may provide valuable insights into predicting treatment outcomes. Further studies are needed to confirm the clinical applicability of PMD as a biomarker for treatment response and to explore its role in other cancer treatments.

## Data Availability

The datasets generated and/or analyzed during the current study are available from the corresponding author upon reasonable request.
